# Adolescents' Exposure to Zero‐Alcohol Advertisements and Attitudes and Consumption Intentions Towards Alcohol: A Cross‐Sectional Study

**DOI:** 10.1111/dar.70125

**Published:** 2026-02-22

**Authors:** Ashlea Bartram, Md Abdul Ahad, Svetlana Bogomolova, Murthy Mittinty, Joanne Dono, Aimee L. Brownbill, Nathan J. Harrison, Jacqueline Garcia, Ivana Glavinic, Mia May, Jacqueline Bowden

**Affiliations:** ^1^ College of Medicine and Public Health, Flinders Health and Medical Research Institute, Flinders University Adelaide Australia; ^2^ National Centre for Education and Training on Addiction Flinders University Adelaide Australia; ^3^ Centre for Social Impact, College of Business, Government and Law Flinders University Adelaide Australia; ^4^ Health Policy Centre South Australian Health and Medical Research Institute Adelaide Australia; ^5^ School of Psychology The University of Adelaide Adelaide Australia; ^6^ Foundation for Alcohol Research and Education Canberra Australia; ^7^ Independent Researcher Adelaide Australia

**Keywords:** adolescents, advertising, alcohol, NoLo, zero‐alcohol

## Abstract

**Introduction:**

Exposure to alcohol advertising is a key influence on adolescent alcohol consumption. Zero‐alcohol drinks (< 0.5% alcohol by volume) resemble and often share brands with alcoholic drinks, so may function as surrogate alcohol marketing. We examined whether exposure to zero‐alcohol advertising was associated with adolescents' attitudes and consumption intentions towards alcohol.

**Methods:**

*N* = 382 Australians aged 15–17 years completed a cross‐sectional online survey where they viewed zero‐alcohol advertisements from four parent alcohol brands and reported past exposure and liking of each advertisement, attitudes and consumption intentions towards alcohol products from the parent brand, general attitudes and consumption intentions towards alcohol, self‐reported location‐based exposure to zero‐alcohol advertising, prior alcohol and zero‐alcohol consumption, and demographics. Associations between exposure and liking, attitudes, and consumption intentions were examined using linear mixed effect models and linear regression.

**Results:**

Adjusting for prior zero‐alcohol and alcohol consumption, gender and parent presence during survey completion, attitudes towards and intentions to consume alcohol from the parent brand were associated with previous zero‐alcohol advertisement exposure (attitudes: *B* = 0.22, *p* = 0.005; intentions: *B* = 0.20, *p* = 0.002) and zero‐alcohol advertisement liking (attitudes: *B* = 1.42, *p* < 0.001; intentions: *B* = 0.67, *p* < 0.001). No associations were found between self‐reported location‐based exposure to zero‐alcohol advertising and general attitudes and consumption intentions towards alcohol.

**Discussion and Conclusions:**

Findings that adolescents who see and like zero‐alcohol advertisements have more favourable attitudes towards and stronger intentions to consume parent alcohol brands suggest that zero‐alcohol advertisements may serve as surrogate alcohol marketing, supporting calls to include them within alcohol advertising regulations.

## Summary

- Zero‐alcohol drinks (typically < 0.5% alcohol by volume) resemble and often share brands with alcoholic drinks.
- Adolescents who see and like zero‐alcohol advertisements featuring a parent alcohol brand report more favourable attitudes towards the parent brand and report stronger intentions to consume full‐strength alcohol products from the parent brand.
- Findings support arguments that zero‐alcohol advertisements serve as surrogate marketing for full‐strength alcohol products and should be included within alcohol advertising regulations.

## 1 Introduction

Alcohol consumption is particularly harmful in adolescence due to its neurodevelopmental effects [1]. Delaying alcohol initiation and reducing consumption among adolescents is a common priority in alcohol policies and strategies globally [2, 3]. One potential lever used by policymakers is to reduce adolescents' exposure to alcohol advertising. There is a well‐established association between frequency of alcohol advertisement exposure and alcohol consumption among adolescents [4, 5, 6, 7]. The effects of exposure to alcohol advertising are cumulative: the more alcohol advertising a young person is exposed to, the more alcohol they consume [8]; this effect is more pronounced for advertisements that adolescents like or find appealing [9]. Adolescents' exposure to alcohol advertising can be reduced through comprehensive restrictions on alcohol advertising—identified by the World Health Organization as one of the most cost‐effective strategies to reduce harms from alcohol [10]. However, jurisdictions have commonly implemented more targeted restrictions, such as bans on alcohol advertising during the hours or in the locations in which young people are most likely to be present [11, 12].

Zero‐alcohol drinks are drinks that resemble alcoholic drinks in taste and packaging design but contain no or very low volumes of alcohol; thresholds vary from country to country and across policy instruments, but a threshold of 0.5% alcohol by volume is common [13]. The majority of these products—more than 4 in 5 of those sold in the United Kingdom in 2022 [14]—are brand extensions from parent alcohol brands, featuring brand names and employing design elements used on full‐strength alcohol products. Advertisements for these products can also share features with advertisements for their alcoholic counterparts [15]. Despite this resemblance, policies regulating the placement and content of alcohol advertising do not always apply to zero‐alcohol drinks. For example, in Ireland, where alcohol advertising is prohibited on public transport, advertising from zero‐alcohol drinks using parent alcohol brands has featured prominently in an apparent case of surrogate marketing [15]. International [16] and Australian [17] alcohol industry self‐regulatory guides explicitly allow zero‐alcohol advertising to include themes that are discouraged in alcohol advertising, such as claims about hydration and health benefits. These themes have been commonly identified among marketing of zero‐alcohol beer in conjunction with sports sponsorships [18]. Norway has introduced brand‐level restrictions on alcohol advertising, ensuring that these restrictions apply to advertising for all products that feature brands used on full‐strength alcohol products—including zero‐alcohol drinks. However, Lithuania had similar proposed policies dismissed by the courts on the basis that there is not sufficient evidence that consumers associate or confuse zero‐alcohol drinks with their alcoholic counterparts [15, 19].

To date there has been limited research assessing associations between zero‐alcohol advertising exposure and attitudes and consumption intentions towards alcohol. However, evidence from the marketing literature on brand extensions—the use of an established brand name to enter a new product category [20] – is informative. This literature shows that exposure to advertisements for an extension product can result in positive consumer attitudes and increased purchase intentions towards both the extension product and the parent brand. This effect is most likely when there is a good fit between a parent brand and an extension product—such as when they are from similar product categories [20, 21, 22]. Thus, it is plausible that advertisements for zero‐alcohol drinks, particularly those using a parent alcohol brand, will also influence attitudes and consumption intentions towards the parent alcohol brand [23]. In addition, given the resemblance to alcohol advertising, exposure to advertising for zero‐alcohol drinks may also influence attitudes and consumption intentions towards alcohol products in general. A study with Taiwanese adolescents has shown that exposure to influencer marketing for zero‐alcohol beer was associated with zero‐alcohol beer consumption. Zero‐alcohol beer consumption was in turn associated with consumption and intentions to consume alcohol, but this study did not examine whether there was a direct association between exposure to zero‐alcohol beer marketing and alcohol consumption and/or intentions to consume alcohol [24]. A similar effect has also been demonstrated for other alcohol brand extension products, such as branded clothing or other merchandise, with ownership of alcohol‐branded merchandise found to be associated with alcohol initiation among adolescents [25, 26, 27].

In this study, we sought to examine direct associations between exposure to zero‐alcohol advertisements on adolescents' attitudes and consumption intentions towards alcohol. We hypothesised that, among adolescent children:

1. Previous exposure to zero‐alcohol advertisements featuring brand extensions from parent alcohol brands will be associated with more favourable (a) attitudes towards and (b) intentions to consume full‐strength alcohol products from the parent alcohol brand, compared to no previous exposure to zero‐alcohol advertisements featuring brand extensions from parent alcohol brands.
2. Liking zero‐alcohol advertisements featuring brand extensions from parent alcohol brands will be associated with more favourable (a) attitudes towards and (b) intentions to consume full‐strength alcohol products from the parent alcohol brand, compared to not liking zero‐alcohol advertisements featuring brand extensions from parent alcohol brands.
3. Exposure to zero‐alcohol advertisements across different locations/media will be associated with more favourable (a) attitudes and (b) consumption intentions towards alcohol products overall, compared to no exposure to zero‐alcohol advertisements across different locations/media.

## 2 Methods

### 2.1 Study Design

The cross‐sectional online panel survey protocol was registered on the Open Science Framework prior to data collection [28].

### 2.2 Participants and Procedure

This study had ethics approval from Flinders University's Human Research Ethics Committee (Project ID: 6681). Participants were 382 adolescents aged 15–17 years who lived in Australia, recruited to participate in a 10‐min survey in September 2024 from online social and market research panel provider ‘Pureprofile’ and reimbursed AU$2. A sample size of 382 was estimated to provide 90% power to detect effects, assuming an effect size of *f* = 0.2 on all outcomes, a correlation of 0.4 between outcomes, and a family‐wise significance level of 0.05 (applying the Bonferroni correction) [29]. Quotas were applied to ensure that the demographic profile of respondents matched the Australian population for gender, state/territory and metropolitan/regional location. Pureprofile has a panel of over 80,000 Australian account holders aged ≥ 18 years who have indicated their willingness to participate in social and market research and provided detailed profile information. Panellists who had previously indicated that they were a parent or guardian of a child aged 15–17 years and were willing for their children to undertake surveys were sent an invitation to for their child to take part in the study via the panel's electronic platform. They were provided with study information and asked if they consented to their child participating in the study. Those who provided consent were asked to provide their device to their child to complete the survey. The child was then also provided with study information and asked to indicate their consent, and confirm that they had their parent or guardian's consent, to participate before completing the survey. To ensure participant understanding, prior to answering any questions about zero‐alcohol drinks participants were provided with a definition (‘Zero alcohol drinks look and taste like an alcoholic drink (e.g., beer, wine, cider, gin and whisky), but **contain no or very little amounts of alcohol** (less than 0.5% alcohol by volume’)) and shown an image with examples of zero‐alcohol drinks available for sale in Australian supermarkets, as used in previous studies with Australian adolescents [30, 31].

### 2.3 Measures

#### 2.3.1 Independent Variables

##### 2.3.1.1 Advertisement‐Based Previous Exposure and Liking

Participants were shown four video‐based advertisements for zero‐alcohol drinks from four parent alcohol brands (see Table S1, for a description of the advertisements). To select advertisements for inclusion, we compiled a list of parent alcohol brands on zero‐alcohol products available for sale at the two largest supermarket chains in Australia and searched the Meta Ad Library (covering advertisements hosted on Instagram and Facebook) and YouTube between January and April 2024 for advertisements for zero‐alcohol products from these brands posted since 2021. To ensure representation of a range of drink types, we selected one spirit/ready to drink advertisement, one wine advertisement and two beer advertisements because beers comprise over 80% of zero‐alcohol sales in Australia [32]. Where multiple eligible advertisements per product category were identified, we selected the most recent general‐audience advertisement from the parent alcohol brands that had run the largest number of alcohol advertisements in Australia on Meta in 2022 (as an indicator of overall volume of advertising) [33]. For one beer brand, the most recent advertisement was targeted at the audience of a motorsport event. We thus chose the next most recent advertisement that appeared to target a more general audience. We included four advertisements to ensure that the total survey length would not exceed 10 min, which is a recommended maximum survey length for adolescents to minimise response fatigue [34].

After viewing each advertisement, participants were asked if they have ever seen this advertisement (response options: ‘Yes’/‘No’/‘Not sure’, dichotomised to ‘Yes’/‘No or not sure’ for analyses), and if they ‘Like this ad’, ‘Have no opinion about this ad’, or ‘Dislike this ad’, dichotomised to ‘Like’ or ‘Neutral/dislike’ for analyses (adapted from [35]).

##### 2.3.1.2 Location‐Based Exposure

Participants were asked if they had seen advertising for zero‐alcohol drinks in a range of media/locations, including television, social media (e.g., TikTok, Facebook, Instagram), internet, at or around supermarkets, in a bottle shop, at sporting events, radio, newspapers, magazines, restaurants/pubs, billboards/posters, or other locations with a dichotomous response option (‘Yes’/‘No’), adapted from [5].

#### 2.3.2 Dependent Variables

##### 2.3.2.1 Attitudes Towards Parent Alcohol Brands

For each of four parent alcohol brands featured in the advertisements, participants were asked to indicate on a 7‐point semantic differential scale the extent to which they find these brands ‘Unappealing/appealing’, ‘Bad/good’, ‘Unpleasant/pleasant’, ‘Favourable/unfavourable’ and ‘Unlikable/likable’, a validated measure of brand attitude [36] that has previously been used with young people [37]. A summary attitude score was created by taking the mean response (Cronbach's *α* = 0.87).

##### 2.3.2.2 Consumption Intentions Towards Alcohol Products From Parent Alcohol Brands

Consumption intentions towards alcohol products from parent alcohol brands were assessed as the mean response to two statements: ‘I would like to drink alcohol products by [brand]’, and ‘I intend to drink alcohol products by [brand]’, with response options ‘Strongly agree = 5’, ‘Agree = 4’, ‘Neither agree or disagree = 3’, ‘Disagree = 2’ and ‘Strongly disagree = 1’, adapted from a measure of purchase intentions [38] that has been previously used with young people [37].

##### 2.3.2.3 Attitudes Towards Alcohol Products

Attitudes towards alcohol products in general were measured as the mean response to five items: ‘Alcoholic drinks are something that I would enjoy’, ‘Alcoholic drinks are something that would make me feel good’, ‘Alcoholic drinks are something that would make a good impression on other people’, ‘Alcoholic drinks are something that would help me to fit in with others’ and ‘Alcoholic drinks would help me to feel accepted’. These items were adapted from the emotional and social subscales of the shortened consumer perceived value scale, a validated measure of consumers' assessment of the utility of a product [39] that has previously been used to measure alcohol attitudes among young people [40]. Participants responded on a 5‐point Likert scale (‘Strongly agree = 5’, ‘Agree = 4’, ‘Neither agree or disagree = 3’, ‘Disagree = 2’ and ‘Strongly disagree = 1’; Cronbach's *α* = 0.87).

##### 2.3.2.4 Consumption Intentions Towards Alcohol Products

The mean response to three items including ‘I would drink alcoholic drinks if they were offered to me’, ‘I would like to drink alcoholic drinks on a regular basis’ and ‘I cannot imagine ever wanting to drink alcoholic drinks’ (reverse coded) was used to assess consumption intentions towards alcoholic products in general, adapted from items previously used to measure intentions to consume beverages among young people [31, 41]. Participants responded on a 5‐point Likert scale (‘Strongly agree = 5’, ‘Agree = 4’, ‘Neither agree or disagree = 3’, ‘Disagree = 2’ and ‘Strongly disagree = 1’; Cronbach's *α* = 0.76).

##### 2.3.2.5 Other Variables

Participants reported age, gender and residential postcode. Postcodes were mapped to the Index of Relative Socioeconomic Disadvantage [42] and Australian Statistical Geography Standard [43] as indicators of socio‐economic status and metropolitan or non‐metropolitan location respectively. To measure zero‐alcohol initiation, participants were asked ‘Have you ever had even a part of a zero‐alcohol drink?’, with response options ‘No’, ‘Yes, just a few sips’, ‘Yes, I have had fewer than 10 zero‐alcohol drinks in my life’ and ‘Yes, I have had 10 or more zero‐alcohol drinks in my life’. Responses were dichotomised to ‘Never a full drink’ (‘No’/‘Yes, just a few sips’) or ‘At least one full drink’ (‘Yes, I have had fewer than 10 zero‐alcohol drinks in my life’/‘Yes, I have had 10 or more zero‐alcohol drinks in my life’) for analyses. The same format was used to measure alcohol initiation (adapted from [44]). Participants also indicated whether their parent was present in the room while they completed the survey (response options: ‘Yes’/‘No’) as this may have influenced responses to questions about alcohol [45].

Independent and dependent variables used to examine location‐based exposure (i.e., location‐based exposure, attitudes and consumption intentions towards alcohol products in general), as well as all other variables except parental presence, were asked prior to independent and dependent variables used to examine advertisement‐based exposure (i.e., advertisement‐based previous exposure and liking, attitudes and consumption intentions towards parent alcohol brands). This was so that exposure to zero‐alcohol advertisements during the survey did not influence responses to the earlier questions.

### 2.4 Analyses

After calculating descriptive statistics for all variables, linear mixed effects models were used to examine associations between previous exposure to and liking of zero‐alcohol advertisements featuring brand extensions and (a) attitudes and (b) consumption intentions towards the brand, with responses clustered within participants: each participant reported advertisement exposure, liking, attitudes, and consumption intentions for each ad/brand. Gender, alcohol initiation, zero‐alcohol initiation, and parental presence during survey completion were included as controls in each model. Associations were considered significant if the 95% confidence intervals of beta coefficients excluded zero. An examination of variance inflation factors suggested that multicollinearity was not present in the adjusted models (all variance inflation factors < 1.1, below the threshold of 5–10 [46]).

Linear regression analyses were used to examine associations between location‐based exposure to zero‐alcohol advertisements and (a) attitudes and (b) consumption intentions towards alcohol. Following the approach of Jones and Magee [5], separate models were created for each location, as well as the total number of locations in which participants reported seeing advertising. Models first included attitudes as a dependent variable followed by consumption intentions. Gender, alcohol initiation, zero‐alcohol initiation, and parental presence were included as control variables in each model. To adjust for family‐wise error, the alpha level for significance for each model was set at 0.05/11 = 0.005 (Bonferroni correction) and associations were considered significant if the 99.5% confidence intervals of beta coefficients excluded zero [47].

All analyses were performed using SPSS version 28 [48]. Analyses followed the pre‐registered protocol [28] with the exception of using a more stringent alpha level to test Hypothesis 3 to adjust for family‐wise error.

## 3 Results

### 3.1 Sample Characteristics

Characteristics of the study sample are presented in Table 1. 52.6% were female, 39.8% were 17 years old and 69.4% lived in a metropolitan location. Participants from the most disadvantaged two quintiles were slightly underrepresented (representing less than 20% of the sample). 29.3% of participants reported that they had previously drunk at least one full drink of alcohol, while 12.3% reported that they had drunk at least one full drink of zero‐alcohol in their lifetime. While completing the survey, 73.6% had a parent present in the same room.

**TABLE 1:** Participant characteristics (*N* = 382).

	*n*	%
Gender		
Male/man/boy	181	47.4
Female/woman/girl	201	52.6
Non‐binary	0	0.0
A different term	0	0.0
Age, years		
15	110	28.8
16	120	31.4
17	152	39.8
Location		
Metropolitan	265	69.4
Non‐metropolitan	117	30.6
Quintile of Relative Socio‐economic Disadvantage		
Quintile 1—Most disadvantaged	60	15.7
Quintile 2	68	17.8
Quintile 3	91	23.8
Quintile 4	80	20.9
Quintile 5—Least disadvantaged	83	21.7
Parental presence during survey completion		
No	101	26.4
Yes	281	73.6
Ever consumed alcohol		
Never a full drink	270	70.7
At least one full drink	112	29.3
Ever consumed zero‐alcohol		
Never a full drink	335	87.7
At least one full drink	47	12.3

### 3.2 Previous Exposure and Liking of Zero‐Alcohol Advertisements From Parent Alcohol Brands

As shown in Table 2, the proportion of participants who reported that they had previously seen each of the four zero‐alcohol advertisements shown ranged from 5.5% to 18.1%. The proportion of participants who reported liking each advertisement ranged from 16.0% to 53.1%. Among participants who did not report liking an advertisement, more participants had no opinion about the ad than disliked the ad.

**TABLE 2:** Previous exposure and liking of zero‐alcohol advertisements (*N* = 382).

	Ad 1 (beer), %	Ad 2 (beer), %	Ad 3 (spirit), %	Ad 4 (wine), %
Previous exposure to zero‐alcohol advertisement				
Yes	18.1	15.7	6.5	5.5
No	78.8	80.9	90.3	92.7
Not sure/don't know	3.1	3.4	3.1	1.8
Liked zero‐alcohol advertisement				
Liked this ad	27.0	53.1	39.8	16.0
Had no opinion about this ad	61.3	31.7	51.3	65.4
Disliked this ad	11.8	15.2	8.9	18.6

Table 3 displays the results of bivariate linear mixed effects models examining associations between previous exposure, liking, and covariates as independent variables and brand attitudes and intentions to consume alcohol from the parent brand featured in each advertisement (each model contained a single independent variable). Consistent with Hypothesis 1, there was a positive association between previous exposure and brand attitudes, with participants who had previously seen the advertisement on average scoring 0.55 points higher on brand attitudes (on a scale from 1 (unfavourable attitude) to 7 (favourable attitude); 95% confidence interval [CI] 0.37, 0.74); similarly, there was a positive association between previous exposure and consumption intentions, with participants who had previously seen the advertisement on average scoring 0.37 points higher on consumption intentions (on a scale from 1 (strongly disagree) to 5 (strongly agree); 95% CI 0.24, 0.50). Consistent with Hypothesis 2, there were positive associations between liking and brand attitudes and consumption intentions, with participants who liked the advertisement on average scoring 1.45 points higher on brand attitudes (95% CI 1.35, 1.56) and 0.70 points higher on consumption intentions (95% CI 0.62, 0.78). Among covariates, there were positive associations between zero‐alcohol initiation and brand attitudes and consumption intentions such that participants who had previously consumed zero‐alcohol drinks on average scored 0.57 points higher on brand attitudes (95% CI 0.20, 0.94) and 0.74 points higher on consumption intentions (95% CI 0.47, 1.00); and a positive association between alcohol initiation and consumption intentions such that participants who had previously consumed alcoholic drinks on average scored 0.21 points higher on consumption intentions (95% CI 0.01, 0.41). There were no significant associations with attitudes or consumption intentions for gender, alcohol initiation or parental presence.

**TABLE 3:** Bivariate linear mixed effects models between independent variables and attitudes/consumption intentions towards the parent alcohol brand (*N* = 1528 observations among 382 participants).

Independent variable	Brand attitude				Consumption intentions			
	Model	B	95% CI	*p*	Model	B	95% CI	*p*
Previous exposure (ref: no)	1.	0.55	0.37, 0.74	< 0.001	7.	0.37	0.24, 0.50	< 0.001
Liking (ref: no/neutral)	2.	1.45	1.35, 1.56	< 0.001	8.	0.70	0.62, 0.78	< 0.001
Gender (ref: female/woman/girl)	3.	−0.04	−0.29, −0.20	0.726	9.	0.05	−0.13, 0.24	0.562
Alcohol initiation (ref: no)	4.	0.14	−0.12, 0.41	0.290	10.	0.21	0.01, 0.41	0.037
Zero‐alcohol initiation (ref: no)	5.	0.57	0.20, 0.94	0.002	11.	0.74	0.47, 1.00	< 0.001
Parental presence (ref: no)	6.	−0.10	−0.37, 0.18	0.494	12.	−0.04	−0.25, 0.17	0.702

*Note:* Each model number represents a separate model with the listed variable included as an independent variable and brand attitude (models 1–6) or intentions to consume alcoholic products from the parent alcohol brand (models 7–12) as dependent variable.

Abbreviation: CI, confidence interval.

As shown in Table 4, after adjusting for gender, alcohol initiation, zero‐alcohol initiation, and parental presence, associations with previous exposure and liking both remained significant, supporting Hypotheses 1 and 2. In the adjusted model, holding all other independent variables constant, previous exposure was associated with a score 0.22 points higher on brand attitudes (95% CI 0.07, 0.38) and 0.20 points higher on consumption intentions (95% CI 0.07, 0.32) while liking the advertisement was associated with a score 1.42 points higher on brand attitudes (95% CI 1.32, 1.53) and 0.67 points higher (95% CI 0.58, 0.75) on consumption intentions. Among covariates, only the association between zero‐alcohol initiation and consumption intentions remained significant (*B* = 0.52, 95% CI 0.27, 0.77).

**TABLE 4:** Linear mixed effects models examining associations between previous advertisement exposure, liking, and attitudes/consumption intentions towards the parent alcohol brand (*N* = 1528 observations among 382 participants).

Fixed effects	Brand attitude			Consumption intentions		
	B	95% CI	*p*	B	95% CI	*p*
Intercept	3.97	3.74, 4.20	< 0.001	2.32	2.13, 2.51	< 0.001
Previous exposure (ref: no)	0.22	0.07, 0.38	0.005	0.20	0.07, 0.32	0.002
Liking (ref: no/neutral)	1.42	1.32, 1.53	< 0.001	0.67	0.58, 0.75	< 0.001
Gender (ref: female/woman/girl)	−0.05	−0.24, 0.15	0.634	0.06	−0.10, 0.22	0.467
Alcohol initiation (ref: no)	0.03	−0.19, 0.24	0.805	0.11	−0.07, 0.29	0.219
Zero‐alcohol initiation (ref: no)	0.20	−0.10, 0.50	0.199	0.52	0.27, 0.77	< 0.001
Parental presence (ref: no)	−0.04	−0.27, 0.18	0.692	−0.14	−0.20, 0.17	0.878

Random effects	Variance	SE	Variance	SE
Participant (intercept)	0.78	0.07	0.55	0.05

Abbreviation: CI, confidence interval.

### 3.3 Exposure to Zero‐Alcohol Advertisements by Location

As shown in Table 5, participants were most likely to report having seen zero‐alcohol advertisements on social media (20.9%) or elsewhere on the internet (18.1%), at or around supermarkets (17.5%), or on television (15.2%); the mean number of locations in which participants reported seeing advertisements was 1.1 (SD = 1.6). In linear regression models adjusting for gender, alcohol initiation, zero‐alcohol initiation, and parental presence, there were no associations between reporting seeing zero‐alcohol advertisements in any specific location and attitudes or consumption intentions towards alcohol, nor were there associations with the number of locations in which participants reported seeing advertisements (Table 5); Hypothesis 3 was thus not supported.

**TABLE 5:** Prevalence of exposure to zero‐alcohol advertisements by location and associations with attitudes and consumption intentions towards alcohol (*N* = 382).

Advertisement medium	%	Attitudes			Consumption intentions		
		B	99.5% CI	*p*	B	99.5% CI	*p*
Social media (e.g., TikTok, Facebook, Instagram)	20.9	−0.06	−0.39, 0.26	0.577	−0.09	−0.37, 0.18	0.345
Internet	18.1	0.03	−0.32, 0.36	0.833	0.05	−0.24, 0.34	0.628
At or around supermarkets	17.5	−0.05	−0.39, 0.30	0.705	−0.12	−0.41, 0.18	0.255
Television	15.2	−0.03	−0.39, 0.33	0.820	−0.01	−0.32, 0.30	0.933
In a bottle shop	10.5	−0.15	−0.57, 0.28	0.327	−0.17	−0.54, 0.19	0.179
Restaurants/pubs	8.4	−0.04	−0.50, 0.43	0.823	−0.08	−0.48, 0.32	0.587
Billboards/posters	5.5	0.03	−0.54, 0.60	0.876	−0.27	−0.75, 0.22	0.119
Radio	4.7	−0.15	−0.77, 0.46	0.478	0.03	−0.50, 0.55	0.891
At sporting events	4.5	0.13	−0.50, 0.75	0.562	0.25	−0.28, 0.79	0.181
Magazines	2.9	0.26	−0.51, 1.02	0.339	−0.01	−0.67, 0.64	0.954
Newspapers	1.8	0.32	−0.64, 1.28	0.350	0.28	−0.55, 1.10	0.343
Number of locations in which zero‐alcohol advertisements seen	*M* = 1.1 (SD = 1.6)	−0.01	−0.09, 0.08	0.793	−0.02	−0.09, 0.06	0.502

*Note:* B = unstandardised regression co‐efficient. Models adjusted for the following covariates: gender, alcohol initiation, zero‐alcohol initiation, parental presence.

Abbreviation: CI, confidence interval.

## 4 Discussion

In the current study, participants who reported previously seeing and liking zero‐alcohol advertisements from parent alcohol brands reported more favourable attitudes towards and stronger intentions to consume alcoholic products from these brands. This is consistent with previous research that has shown an association between exposure to [4, 5, 6, 7] and liking of [9] alcohol advertising and adolescent alcohol consumption. As this was an observational study, this finding does not provide causal evidence that exposure to and liking of zero‐alcohol advertisements leads to increased intentions to consume alcohol among adolescents. Other research has suggested that the association between alcohol advertising exposure and alcohol consumption is bidirectional, with exposure to alcohol advertising prompting adolescents to consume alcohol while adolescents who have initiated alcohol consumption also notice more alcohol (or alcohol‐like) advertising [49]. Although we adjusted for alcohol and zero‐alcohol initiation in our analysis, it is nonetheless possible that adolescents who already held favourable attitudes towards and intended to drink alcoholic products from the parent alcohol brands included in this study were more likely to report previous exposure to and liking of zero‐alcohol advertisements from these brands. Since digital marketing on social media platforms is typically algorithmic and data driven, the advertisements may have been more likely to be shown to people whose prior use of the platform suggests they had an interest in alcohol or zero‐alcohol drinks [50]. Nonetheless, our findings corroborate other evidence that zero‐alcohol advertisements may act as surrogate marketing for alcohol products, including theoretical arguments based on the strong visual resemblance and use of parent alcohol brands [15, 23], policy analyses [51], experimental findings that zero‐alcohol drinks prompt adolescents to think of alcohol [30], and studies of parent and adolescent perceptions of zero‐alcohol drinks [31, 52].

Location‐based measures of exposure to zero‐alcohol advertisements were not associated with attitudes and consumption intentions towards alcohol in this study. This contrasts with previous research that has found that exposure to alcohol advertisements in a range of locations including magazines, newspapers, on the internet, billboards, bottle shops, and bars was associated with alcohol initiation among adolescents [5]. The lack of association for zero‐alcohol advertisements may reflect limitations of the self‐reported measure of exposure, which required that participants recognised that an advertisement they were viewing was for a zero‐alcohol drink and not an alcoholic drink and were able to recall this while completing the survey. Given the strong resemblance between zero‐alcohol and alcohol advertisements, it is possible that adolescents do not always notice whether an advertisement they see is for a zero‐alcohol or alcohol‐containing product, or may be confused about whether a zero‐ or full‐strength alcohol product is being advertised. Indeed, adolescents have been shown to categorise zero‐alcohol drinks as alcoholic beverages [30]. The prevalence of exposure to zero‐alcohol advertisements in different locations may thus be underestimated in this study, compromising the study's capacity to test for associations between exposure and attitudes and consumption intentions towards alcohol.

### 4.1 Strengths, Limitations and Future Directions

Strengths of this study included its pre‐registered analysis plan, use of two alternative measures of advertisement exposure to examine potential effects of both brand extension advertising and zero‐alcohol advertising more generally, and involvement of a diverse sample of Australian adolescents that was demographically similar to the Australian population in terms of the reported characteristics. The sample was not representative of all adolescents as it was recruited from an online panel; however, it was not designed to be generalisable as the priority was to test the hypothesised relationships within a hard‐to‐reach population group. As a condition of our ethics approval, we required parental consent from all participants, which in practise led to the majority of respondents completing the survey while a parent was present. Although we adjusted for parental presence in analyses, it may still have affected responses; the direction of this effect is difficult to predict as it may have influenced responses to both outcome measures (attitudes, consumption intentions towards alcohol) and covariates (prior consumption). Our measures of exposure were limited in that they relied on self‐report and did not capture number or frequency of exposure. As we did not have a direct measure of previous exposure, we were also not able to account for exposure to other zero‐alcohol advertisements, either from the same or other brands, in our analyses of advertisement‐based exposure and liking. As discussed above, our design was cross‐sectional, limiting our capacity to infer causality. Future studies could use longitudinal designs and assess exposure more directly, for example by working with participants to record their exposure via ecological momentary assessment designs [53] or through confirmed attendance at events or viewing of broadcasts known to feature (zero‐)alcohol advertising [54]. These studies could also examine the extent to which exposure to or liking of zero‐alcohol advertisements from specific parent brands may influence intentions to consume alcohol in general as well as alcoholic products from the parent brand.

### 4.2 Policy Implications

These findings provide corroborating evidence to support concerns that zero‐alcohol advertising may serve as surrogate marketing for alcohol products. The current study, in conjunction with prior research and commentary [15, 23, 30, 31, 51, 52], suggests that there is a need to extend restrictions that limit adolescents' exposure to alcohol advertising to cover zero‐alcohol drinks and minimise the extent to which adolescents see alcohol‐like advertising that they find appealing. The findings suggest that it is insufficient to limit exposure only to advertisements that hold ‘strong or evident’ [17] or ‘primary’ [16] appeal to minors, as is currently specified in many self‐regulatory codes: all advertisements tested held appeal for at least some minors in the sample, despite containing no youth‐specific content. Rather, mandatory regulations on the locations in which alcohol and zero‐alcohol advertisements can be placed (e.g., as implemented under France's Loi Evin [11]), or comprehensive, brand‐level restrictions on alcohol advertising (as implemented in Norway [55]) may be needed to meaningfully reduce adolescents' exposure to alcohol‐like advertising.

## 5 Conclusion

Findings that adolescents who see and like zero‐alcohol advertisements from parent alcohol brands have more favourable attitudes towards and stronger intentions to consume full‐strength alcohol products from those brands support concerns that these advertisements serve as surrogate marketing for full‐strength alcohol and should be included within the scope of alcohol advertising regulations.

## Author Contributions

Each author certifies that their contribution to this work meets the standards of the International Committee of Medical Journal Editors.

## Funding

This research was funded by the Channel 7 Children's Research Foundation (Project Reference: 24‐36090441). AB, MAA and JB receive funding from the Australian Government Department of Health and Aged Care to support research regarding alcohol and other drugs (4‐IN4X1RZ).

## Conflicts of Interest

N.J.H. is a Guest Editor for the ‘Understanding the emergence, availability and consumption trends of no and low alcohol (‘NoLos’) beverage products’ Special Section in *Drug and Alcohol Review*. To minimise bias, they were excluded from all editorial decision‐making related to this manuscript. Remaining authors have no conflicts of interest to declare.

## Supporting information


**Table S1:** Description of advertisements.

## Data Availability

The data that support the findings of this study are available from the corresponding author upon reasonable request.
