# Supplementary material for: Adolescents' Exposure to Zero‐Alcohol Advertisements and Attitudes and Consumption Intentions Towards Alcohol: A Cross‐Sectional Study
Source: Drug Alcohol Rev. 2026 Feb 22;45(2):e70125. doi: 10.1111/dar.70125 (PMC12925609; doi:10.1111/dar.70125)
Supplement: Supplementary file 1 — Table S1: Description of advertisements. [file DAR-45-0-s001.docx]

**Supporting Information**

**Table S1. Description of advertisements**

| **Advertisement** | **Product** | **Tag line** | **Description** |
| --- | --- | --- | --- |
| Ad 1 (beer) | Great Northern Zero | Full taste with zero alcohol. For doing what you love in the great outdoors. | Depicts a bottle of the product in the foreground while zooming out on an image of people fishing from a boat in the background. |
| Ad 2 (beer) | Heineken 0.0 | Cheers with no alcohol. Now you can. | Depicts a series of scenarios in which people raise a toast holding alcoholic drinks while a person holding a non-alcoholic drink is excluded. Ad concludes with a scenario in which a person raises a toast using the product. |
| Ad 3 (spirit) | Gordons Alcohol Free 0.0% | 0% Alcohol, 100% Gordon’s. Shall We? | Depicts a glass containing a clear, carbonated drink, ice, a lime slice, and a straw, then zooms out to show a bottle of the product and a second glass. |
| Ad 4 (wine) | Hardys Zero Shiraz | Zero alcohol, same great taste | Depicts a person’s hand sprinkling cheese on pizza dough next to a bottle and glass of the product, while another person pours a glass of the product in the background |
